# National Tuberculosis Genotyping and Surveillance Network: Design and Methods

**DOI:** 10.3201/eid0811.020296

**Published:** 2002-11

**Authors:** Jack T. Crawford, Christopher R. Braden, Barbara A. Schable, Ida M. Onorato

**Affiliations:** * Centers for Disease Control and Prevention, Atlanta, Georgia, USA

**Keywords:** *Mycobacterium tuberculosis*, strain typing, surveillance

## Abstract

The National Tuberculosis Genotyping and Surveillance Network was established in 1996 to perform a 5-year, prospective study of the usefulness of genotyping *Mycobacterium tuberculosis* isolates to tuberculosis control programs. Seven sentinel sites identified all new cases of tuberculosis, collected information on patients and contacts, and obtained patient isolates. Seven genotyping laboratories performed DNA fingerprinting analysis by the international standard IS*6110* method. BioImage Whole Band Analyzer software was used to analyze patterns, and distinct patterns were assigned unique designations. Isolates with six or fewer bands on IS*6110* patterns were also spoligotyped. Patient data and genotyping designations were entered in a relational database and merged with selected variables from the national surveillance database. In two related databases, we compiled the results of routine contact investigations and the results of investigations of the relationships of patients who had isolates with matching genotypes. We describe the methods used in the study.

**Figure:**
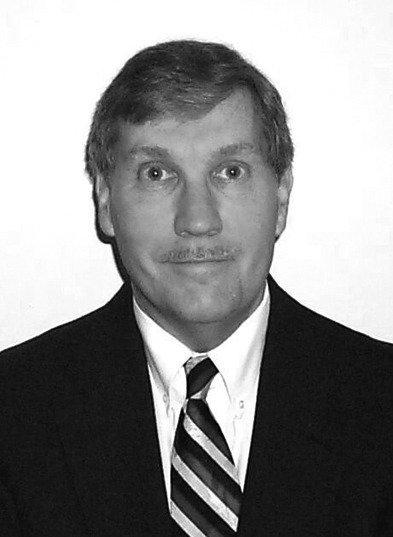
**Jack T. Crawford, Guest Editor.** Dr. Crawford is chief of the Immunology and Molecular Pathogenesis Section, Tuberculosis/Mycobacteriology Branch, Division of AIDS, STD, and TB Laboratory Research, Centers for Disease Control and Prevention. His research interests include application of molecular methods to epidemiology and diagnostics of mycobacterial diseases.

Molecular characterization of strains of *Mycobacterium tuberculosis* has been used for more than a decade to study the epidemiology of tuberculosis (TB). DNA fingerprinting, with IS*6110* as a probe, has been used successfully to trace transmission of *M. tuberculosis* in outbreaks, confirm laboratory cross-contamination, and identify risk factors for disease among populations of patients with TB (1). Before 1993, epidemiologic studies that used molecular characterization of *M. tuberculosis* were focused on populations, such as persons who were HIV positive or were in nosocomial settings (2–4). The standardization of methods provided an opportunity to examine *M. tuberculosis* strains from TB patients throughout the world and encompassed a variety of settings and populations (5).

In response to the upsurge of TB cases in the United States, the Centers for Disease Control and Prevention (CDC) funded regional laboratories to provide fingerprinting services to support TB control programs in outbreak investigations and to conduct studies on using DNA fingerprinting in TB epidemiology and control. This network was expanded to include sentinel surveillance sampling in 1996, when CDC established the National Tuberculosis Genotyping and Surveillance Network as a 5-year project. The genotyping network involved seven sentinel surveillance sites^1^ that were paired with seven genotyping laboratories.^2^ The historical background and objectives of the genotyping network have been discussed elsewhere (6). In this paper, we summarize the specific project methods.

## Network Participants

The sentinel surveillance sites included the states of Arkansas, Maryland, Massachusetts, Michigan, and New Jersey; six counties in California surrounding San Francisco (Alameda, Contra Costa, Marin, San Mateo, Santa Clara, and Solano); and four counties in Texas representing two regions of the state with distinct demographics (Dallas, Tarrant, Cameron, and Hidalgo) (Figure). State departments of health TB-control programs conducted the project activities for each site. The seven laboratories provided DNA fingerprint analyses of *M. tuberculosis* isolates for their sentinel surveillance sites. In addition, genotyping network laboratories were assigned regions for which they provided genotype analysis of *M. tuberculosis* isolates in support of investigations by state departments of health. These isolates and associated patients were not included in the network databases. Regions were delineated on the basis of the approximate number of patients with TB in the regions; regions did not follow the boundaries of the U.S. Public Health Service Regions.

**Figure:**
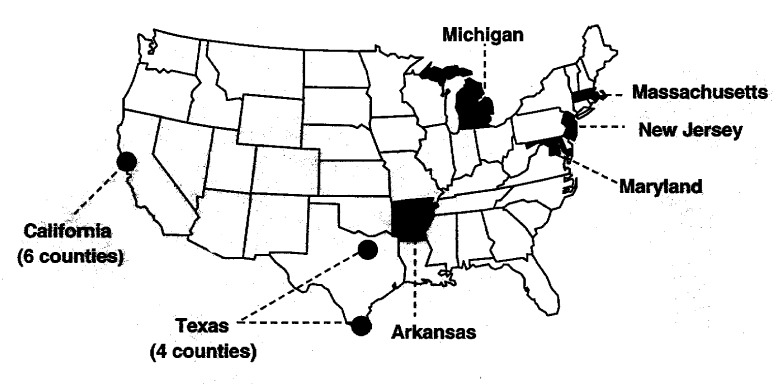
Map showing locations of the sentinel surveillance sites in the National Tuberculosis Genotyping and Surveillance Network, United States.

## Case Finding and Isolate Collection

Sources of information to identify TB cases within surveillance sites included hospital and clinic records from all facilities serving patients in the area, records of laboratories performing mycobacteriology services, hospital ICD-9CM discharge codes for TB, pharmacy records for prescriptions of anti-TB drugs, medical examiners’ or coroners’ records, death certificates, and AIDS surveillance reports that listed a diagnosis of TB. Culture-positive, verified TB cases reported from January 1996 through December 2000 for national TB surveillance to CDC (reported on the Report of a Verified Case of Tuberculosis) were included as sentinel surveillance cases. Patients with recurrent TB occurring >1 year after completion of therapy are considered new case-patients; a small number of such cases were included. Patients later identified as residing outside of the surveillance area and those with positive cultures as the result of laboratory cross-contamination were excluded.

Every effort was made to acquire an isolate from each study patient. This task was difficult because many clinical samples were sent for culturing to laboratories other than the state public health laboratory, including hospital and commercial laboratories, some out of state. Isolates identified as *M. tuberculosis* complex, i.e., *M. tuberculosis*, *M. bovis,* and *M. africanum,* were included in the study. Isolates were sent to the assigned genotyping laboratory for typing. Stocks of all isolates were maintained at –70°C, and all isolates were transferred to CDC for long-term storage and future studies.

## Laboratory Protocols

All National Tuberculosis Genotyping and Surveillance Network laboratories used standardized protocols. Isolates were typed by the international standard IS*6110* method, i.e., digestion of DNA with endonuclease *Pvu*II and hybridization with a probe containing the right end of IS*6110* (5). Growth was harvested directly from Lowenstein-Jensen slants, or isolates were subcultured in Middlebrook 7H9 broth. Cell suspensions were heat-killed before DNA was extracted. DNA was digested with *Pvu*II and run on a 20-cm, 1% agarose gel without ethidium bromide. Electrophoresis was conducted at 100 V for a short time to allow the DNA to enter the gel and then overnight at lower voltage until the dye front had run approximately 17 cm. An external size standard was run in wells 1, 10, and 20 on a 20-well gel. The gel was stained with ethidium bromide and photographed on a UV transilluminator to check for quality, including complete digestion of the DNA samples, degradation of DNA, and uneven running or smiling of the gel. Gels were blotted and hybridized in the standard fashion with chemiluminescent probes (ECL kit, Amersham Biosciences, Piscataway, NJ) and x-ray film, with the exception of one laboratory that used ^32^P-labeled probes and a phosphoimager to generate direct digital images.

Two different size standards were used. Initially, total genomic DNA from *M. tuberculosis* strain 2650, provided by the genotyping laboratory at the Public Health Research Institute, New Jersey, was used. This standard was replaced by a recombinant size standard prepared at CDC; this standard contains 25 fragments of sequences, ranging in size from 700 to 15,000 bp. All fragments contain at least one copy of a 500-bp segment of the right side of IS*6110* and are detected by the IS*6110* probe. When the recombinant standard was available, the sizes of the fragments in the original 2650 standard were determined relative to the new standard, and these sizes were used to reanalyze all of the prior images containing the 2650 standard.

## Computer-Assisted Pattern Analysis

IS*6110* patterns were scanned and then analyzed by regional laboratory personnel by using BioImage Whole Band Analyzer software, version 3.4 (BioImage, Ann Arbor, MI), a UNIX-based program run on Sun workstations (Sun Microsystems, Inc., Santa Clara, CA). This version of the software included enhancements developed specifically for the TB genotyping network. The software performed automatic band identification, but each image was visually evaluated and edited, as needed. The greatest difficulty was deciding if bands of greater than average width or intensity represented single or multiple fragments. The protocol called for assigning multiple bands only when separate bands were clearly evident, which often required observation of multiple exposures of the original films. Bands with lower than average intensity were counted only if the peak height in the scanned image was at least two thirds that of adjacent bands. The logarithmic scale method was used to size bands.

For pattern matching, BioImage software used the Jaccard coefficient of similarity between two patterns, A and B (100 x number of matched bands [number of bands in A + number of bands in B – number of matched bands]). Pairs of patterns were compared for matching bands by a deviation of ±2.5% for molecular weight (not distance migrated). All matches were verified by visual comparisons. Only patterns that were identical, i.e., had the same number of bands of the same sizes (±2.5%), were given the same designation.

Each regional laboratory maintained a database of fingerprint patterns for all isolates from the laboratory’s sentinel site. New patterns were submitted to CDC for assignment of a genotyping network database designation. Any subsequent isolates with the identical fingerprint pattern were assigned the same designation and were not submitted to CDC. Files of single gel lanes were transmitted to a secure site at CDC. These files contained the original scanned image and the data file of calculated band sizes. In these images, bands included in the analysis were highlighted, and the locations of the bands in the standard were indicated. The database manager examined the images to determine if they were of acceptable quality but did not make changes in the submitted files. Apparent discrepancies were referred to the submitting laboratory for resolution, and the final decision about band identification rested with the laboratory that performed the analysis and had the original films. Submitted patterns were compared to the patterns in the existing TB genotyping network database by using the parameters described above. New patterns were added to the database and assigned consecutive 5-digit designations. When a submitted pattern matched a pattern already in the database, the submitted pattern was reported back with the existing designation. Thus, the TB genotyping network fingerprint database contained single examples of all distinct patterns from all seven regional laboratories during the 5-year study period. The designation 00000 was assigned to isolates having zero copies of IS*6110*, i.e., gave no bands. Some patterns were dropped from the study, e.g., if the isolate was the result of cross-contamination or the patient was from outside the study area. These designations were not reused; therefore, the total number of patterns (6,128) in the final database is lower than the highest designation (pattern 07193).

## Secondary Typing

During the early part of the study, polymorphic GC-rich sequence (PGRS) typing, also referred to as pTBN12 (7), was used for secondary typing. However, this method was difficult to standardize, and spoligotyping (spacer oligotyping) was used for secondary typing (8). Isolates with six or fewer bands on IS*6110* typing, including most isolates that had been typed previously by using PGRS, were spoligotyped by using a standard method (9). The spoligotypes were recorded by using a 15-digit octal code to represent the binary result for the 43 spacers (10). Briefly, the 43-digit binary result, representing the 43 spacers (where 1=positive, 0=negative), was divided into 14 sets of 3 digits (spacers 1–42) plus 1 additional digit (spacer 43). Each 3-digit set was converted to octal code (000=0, 001=1, 010=2, 011=3, 100=4, 101=5, 110=6, and 111=7) with the final digit remaining either 1 or 0. This scheme yielded a 15-digit octal designation. To simplify database entries, the CDC database manager assigned consecutive arbitrary 4-digit designations to the octal designations.

## Quality Assurance

Several sets of isolates were prepared at CDC and distributed to the typing laboratories for fingerprinting and analysis. Their results and results of typing at CDC were analyzed to determine reproducibility. Because such challenge sets may receive special handling, a second approach was used in the final 3 years of the project. For each laboratory, the database manager selected IS*6110* patterns at random from those previously submitted by that laboratory to the CDC database. The corresponding isolates, 10 per year per laboratory, were sent to CDC for typing, and results were compared to prior patterns.

## Epidemiologic Databases

Sentinel surveillance sites each maintained a database of their patients and patient isolate genotype designations in Epi-Info 6.04d software (case file) (11). Patients were identified by their state surveillance case numbers. Complete files from each sentinel site were transmitted to CDC on a bimonthly basis and concatenated. These data were merged with select variables from the national TB surveillance database (SURVS-TB and TIMS) in an SAS dataset (SAS Institute, Inc., Cary, NC).

For each sentinel surveillance patient, data were collected concerning any TB case-patients identified as epidemiologically related through routine contact investigations conducted as part of TB-control programs. Epidemiologic connections were defined as direct exposure to an infectious TB patient (e.g., named contacts) or a circumstance in which patients were in the same location at the same time (e.g., the same jail). Related cases identified though routine contact investigations were included in the study if they had been diagnosed on or after January 1, 1990. Data collected for each related case-patient included the following: direction of transmission (source, secondary); type of relationship (household, nonhousehold relatives or friends, co-worker, common source); type of setting of exposure (e.g., correctional facility, school or day-care center, co-worker, emergency shelter, group quarters, hospital, nursing home, other long-term care facility); and date that the epidemiologic relationship was discovered. Information for each related case identified through routine contact investigation was entered into a supplemental Epi-Info file, which was also routinely submitted to CDC. This database was merged with the case file data in an SAS dataset.

## Genotype Cluster Investigation

Case-patients with isolates demonstrating indistinguishable genotypes, i.e., the same IS*6110*-pattern designation for isolates with more than six IS*6110* bands or the same IS*6110* pattern and spoligotype for isolates with six or fewer IS*6110* bands, were defined as genotype clusters. All cases in clusters required review of epidemiologic information obtained from contact investigations before isolate genotyping to determine known epidemiologic connections. If one or more patients in a cluster had no known epidemiologic connections with others in the cluster, a cluster investigation was initiated. Patients participated with informed consent. Cluster investigations were conducted prospectively from January 1, 1998, to December 31, 2000. Data from medical record reviews and interviews with the patients were collected by using standardized forms. Information collected from interviews included demographics, TB medical history, any TB exposures, and at least a 2-year history for residence and social, work, and recreational activities. Information collected from medical records and interviews was compared for patients in each cluster to identify epidemiologic connections. The results of cluster investigations were entered into a third Epi-Info file. This file also contained related positive skin-test results, date of initial skin tests, direction of transmission, types of relationships, exposure settings, start dates of likely exposure, and information used to identify relationships (record review, routine interview, contact investigation, and isolate genotyping information). Data were submitted to CDC and converted into an SAS dataset for future analyses.

## Weaknesses in Study Design

From the beginning of the project, we recognized that compiling results from multiple laboratories would be challenging because of the variability in the IS*6110* fingerprinting method. However, assigning a designation to the IS*6110* pattern for each isolate was necessary to allow entry of the result into the epidemiologic database. Two flaws in the design of the national fingerprint database were identified during the project; both primarily affected the analysis of isolates with low-copy numbers for IS*6110.* The first flaw arose from the computer algorithm for pattern matching. A newly submitted pattern (B) was considered distinct from an existing pattern (A) if any band in the pattern differed by more than the allowed 2.5% variation in size. However, a third pattern with a band of intermediate size can match both A and B. Thus, the software could identify a set of patterns that matched at 100%, even though some individual pairs within the set did not match. The second flaw was procedural. When a laboratory submitted a pattern that matched one already in the database, the existing designation was reported back to that laboratory. In such situations, the submitted image became the reference in that laboratory’s database for that pattern designation. In retrospect, we realize that more consistent results would have been obtained if we had transmitted the prototype image from the national database to the regional laboratories so that everyone would have been using the same patterns for matching, i.e., the same set of band sizes.

Quality assurance results (12) highlighted the need for experienced judgment in evaluating and editing patterns and matching results. In addition, isolates with few copies of IS*6110* and common spoligotypes may not be well discriminated with these genotyping methods. The subjective nature of genotype interpretation and lack of specificity for some isolates may have resulted in some patients being misclassified as clustered or not.

Determination of epidemiologic connections based on routine contact investigations was problematic. Methods used by different jurisdictions varied considerably, as did the completeness of contact investigations. Therefore, interpretation and comparison of the proportion of cases with epidemiologic connections established by contact investigations among the sentinel sites should be done with caution. Genotype cluster investigations proved particularly difficult in several situations. Though cluster investigations were conducted prospectively, some genotype results were delayed for prolonged periods. Patients who had completed therapy were often difficult to locate for interviews. Delay in identifying clusters also occurred when a prolonged period had elapsed between diagnoses and cultures of patient specimens; delays also varied among the sites. The success of identifying epidemiologic links among clustered patients in these circumstances was limited.

## Footnotes

1 Sentinel surveillance sites and personnel: 1) Arkansas Department of Health: Joseph Bates, Donald Cunningham, Kashef Ijaz, Bill Starrett, William Stead, and Frank Wilson; 2) California Department of Health Services: Rocio Agraz, Dan Chin, Jennifer Flood, Peter Oh, Sarah Royce, and Sumi Sun; 3) Maryland Department of Health and Mental Hygiene: Wendy Cronin, Jonathan Golub, David Hooper, Nancy Hooper, Monica Lathan, Leonard Mukasa, and Jafar Razeq; 4) Massachusetts Department of Public Health: Edward Corkren, Paul Elvin, Sue Etkind, Ann Miller, Edward Nardell, Jill Northrup, Alissa Scharf, Sharon Sharnprapai, Alexander Sloutsky, Robert Suruki, and Debra Thimas; 5) Michigan Department of Community Health: Bernie Benecke, Matthew Boulton, Norm Keon, Michael Kucab, Dennis Minnice, Jolynn Montgomery, and Charles Williams II; 6) New Jersey Department of Health and Senior Services: Nancy Buono, Mary Dillon, Zary Liu, Marcia Localio, Christian Nwigwe, Felicidad Santos, Mary Ellen Schulman, and Kenneth Shilkret; 7) Texas Department of Health: Dennis Ashworth, Dale Dingley Jr., Miguel Garza, Jeff Taylor, and Patricia Thickstun.

 2 Genotyping laboratories and personnel: 1) Alabama Department of Public Health and University of Alabama, Birmingham: William Benjamin Jr., Nancy Dunlap, Donna Hafner, Nancy Keenan, Kerry Lok, Donna Mulcahy, Virginia Pruitt, and Nancy Robinson; 2) Central Arkansas Veterans Healthcare Services: M. Donald Cave, Kathleen Eisenach, Ndingsa Fomukong, and ZhenhuaYang; 3) California Department of Health Services: Jason Coloma, Jonna De Leon, Edward Desmond, Athena Fye, Marguerite Griffith, Melvin Javonillo, Travis Jobe, Rose Longoria, Cynthia Sanders, and Lourdes Seli; 4) Michigan Department of Community Health: Dale Berry, Steve Church, Stephen Dietrich, Frances Pouch Downes, Jeffrey Massey, Teresa Miller, Laura Mosher, and Patricia Somsel; 5) New York State Health Department,Wadsworth Center: Jeff Driscoll, Robert Jovall, Mike McGarry, Max Salfinger, and Harry Taber; 6) Public Health Research Institute, New York: Pablo Bifani, Barry Kreiswirth, Natalia Kurepina, Barun Mathema, Soraya Moghazeh, Alex Ravikovitch, and Elena Shashkina; and 7) Texas Center for Infectious Diseases: Yvonne Camarce, Rebecca Cox, J. Seb Gillette, Oscar Gonzalez, Kenneth Jost, D. Mitch Magee, Teresa Quitugua, Ishmael I. Rosas, and Jeffery Taylor.
